# Redefining Dual Antiplatelet Strategies After Acute Coronary Syndrome: Insights from Recent RCTs

**DOI:** 10.3390/jcm15072472

**Published:** 2026-03-24

**Authors:** Maggie He, Joseph Magdy, Maryam Aziz, Jun Tan, Arka Das, Stephen B. Wheatcroft, Heerajnarain Bulluck

**Affiliations:** 1 Yorkshire Heart Centre, Leeds General Infirmary, Leeds Teaching Hospitals NHS Trust, Leeds LS1 3EX, UK; maggie.h117@gmail.com (M.H.);; 2 School of Medicine and Dentistry, Griffith University, Gold Coast 4215, Australia; 3 School of Medicine, Worsley Building, Clarendon Way, University of Leeds, Leeds LS2 9JT, UK; 4 Leeds Institute of Cardiovascular and Metabolic Medicine, University of Leeds, Leeds LS2 9JT, UK

**Keywords:** dual antiplatelet therapy, acute coronary syndrome, P2Y12 inhibitor, bleeding risk, abbreviated DAPT, risk stratification

## Abstract

For nearly two decades, 12 months of dual antiplatelet therapy (DAPT) after acute coronary syndrome (ACS) has been the standard recommendation. Recent evidence suggests that abbreviated DAPT durations may reduce bleeding without compromising ischemic protection in selected patients. This review synthesizes randomized controlled trials, meta-analyses, and guideline updates published between 2023 and 2025, evaluating abbreviated DAPT strategies after ACS with percutaneous coronary intervention. Immediate aspirin withdrawal after PCI increased early stent thrombosis in NEO-MINDSET and STOPDAPT-3. One-month DAPT followed by ticagrelor monotherapy reduced bleeding without increasing ischemic events in ULTIMATE-DAPT and T-PASS. Three-month strategies demonstrated the most consistent safety profile, with TWILIGHT showing 50% bleeding reduction without increased death, myocardial infarction, or stroke (noting that TWILIGHT included 35% chronic coronary syndrome patients). Clopidogrel monotherapy after abbreviated DAPT increased myocardial infarction in STOPDAPT-2 ACS, highlighting the importance of potent P2Y12 inhibition. Meta-analyses confirmed bleeding reductions with early P2Y12 inhibitor monotherapy across broader populations, though benefits were more pronounced in East Asian cohorts. Abbreviated DAPT strategies offer personalized alternatives to standard 12-month therapy. Three-month DAPT followed by ticagrelor monotherapy represents a reasonable and evidence-supported strategy in selected patients with ACS. Risk stratification tools and individual patient factors should guide therapy duration decisions.

## 1. Introduction and Scope

Acute coronary syndrome (ACS) remains the leading cause of death and morbidity worldwide, particularly in low- and middle-income countries. This is despite decreasing age-standardized rates of ischemic heart disease (IHD) in high-income regions attributable to major medical advances in revascularization, primary and secondary prevention. These gains, however, are likely offset by global population growth and ageing [1,2]. ACS encompasses a spectrum of clinical presentations, from unstable angina through non-ST-elevation myocardial infarction (NSTEMI) to ST-segment-elevation myocardial infarction (STEMI). Contemporary classifications distinguish between Type 1 myocardial infarction (MI), characterized by atherothrombotic plaque rupture or erosion, and other forms of myocardial injury. The pathophysiology of these syndromes is all attributed to differing degrees of coronary plaque disruption, thrombosis and consequent myocardial ischemia, often in the setting of platelet activation and aggregation [3].

Percutaneous coronary intervention (PCI) mitigates, but does not eliminate, residual risk of coronary ischemia by restoring vessel patency. Patients remain susceptible to early and late stent-related events, such as stent thrombosis, non-culprit lesion progression, and recurrent atherothrombotic events [4,5,6]. Therefore, antiplatelet therapy plays an important role in reducing recurrent ischemic events. For nearly two decades, dual antiplatelet therapy (DAPT) for 12 months after ACS with a potent P2Y12 inhibitor (such as ticagrelor or prasugrel) has been endorsed as a Class I recommendation by the American College of Cardiology Foundation (ACC)/American Heart Association (AHA) and European Society of Cardiology (ESC) guidelines [4,7]. The evidence behind this approach has been based on earlier randomized trials, showing a net reduction in ischemic events from prolonged DAPT [8,9,10] at the risk of increased bleeding [6]. This default strategy has been widely adopted in patients without prohibitive bleeding risk.

However, this is an evolving field, and between 2023 and 2025, there has been a growing body of evidence in randomized controlled trials, pooled patient-level analyses, and meta-analyses that have redefined this paradigm. Several themes have emerged, including the potential safety of early aspirin withdrawal with continuation of P2Y12-inhibitor monotherapy, the growing evidence for abbreviated DAPT durations of 1–6 months, and the expanding role of risk-stratification tools that aim to balance bleeding and ischemic hazards more effectively than a uniform, “one-size-fits-all” recommendation.

The purpose of this review is to synthesize evidence published and presented predominantly between 2023 and 2025—including late-breaking randomized controlled trials, major meta-analyses, and guideline updates—to clarify how these data may reshape practical, risk-stratified antiplatelet strategies after ACS. Our focus is on the nuanced application of these findings across diverse clinical scenarios, recognizing that the optimal antithrombotic approach increasingly depends on the interplay between patient-level vulnerability, procedural complexity, and the dynamic trajectory of ischemic and bleeding risk.

### Literature Search and Study Selection

A systematic electronic search was conducted in PubMed, EMBASE, and the Cochrane Central Register of Controlled Trials (CENTRAL) from January 2023 to January 2026 using the following search terms: ‘dual antiplatelet therapy’, ‘DAPT’, ‘abbreviated DAPT’, ‘P2Y12 inhibitor monotherapy’, ‘acute coronary syndrome’, ‘ACS’, ‘percutaneous coronary intervention’, and ‘PCI’. Conference abstracts from ACC, ESC, and TCT meetings 2023–2025 were included, where peer-reviewed publications were pending. Non-English studies and unreviewed preprints were excluded. Studies eligible for inclusion were randomized controlled trials, pre-specified subgroup or pooled patient-level analyses, network or pairwise meta-analyses, and major guideline documents evaluating DAPT strategies after ACS with PCI.

## 2. Pathophysiology and Pharmacology

### 2.1. Plaque Disruption and Platelet Biology

ACS typically arises when atherosclerotic plaque is disrupted, for instance, in plaque rupture, plaque erosion, or calcified nodules. This exposes highly thrombogenic materials, including subendothelial collagen, von Willebrand factor, and tissue factor, which lead to platelet adhesion, activation, and aggregation [11,12,13,14]. Platelet activation triggers the release of adenosine diphosphate (ADP) and thromboxane A_2_ (TXA_2_), amplifying aggregation and recruitment. GPIIb/IIIa receptor activation leads to fibrinogen crosslinking between adjacent platelets, forming the structural core of the platelet-rich thrombus. This aggregation step represents the final common pathway of platelet activation and is mechanistically distinct from, though dependent upon, the upstream activation signals mediated by ADP, TXA_2_, and collagen [13,14,15].

These events unfold rapidly. The early dominance of platelet-driven thrombosis in ACS explains why multiple therapeutic nodes—COX-1 inhibition, P2Y12 receptor blockade, and in some cases GPIIb/IIIa inhibition—have clinical relevance. Blocking platelet activation at one or more signaling points remains central to preventing the evolution of a transient plaque injury into an occlusive coronary thrombus.

### 2.2. Antiplatelet Agents—Mechanisms and Clinical Implications

**Aspirin** irreversibly inhibits COX-1 in platelets, preventing TXA_2_ synthesis for the platelet lifespan. Its antithrombotic effect is dose-dependent for COX-inhibition, and low-dose aspirin (75–100 mg daily) provides near-complete platelet COX-1 blockade with an acceptable safety profile [16].

**P2Y12 receptor inhibitors** block ADP-mediated platelet activation and aggregation with varying onset, potency, and reversibility [10,17,18,19,20]:

- **Clopidogrel** is an oral thienopyridine prodrug requiring hepatic bioactivation predominantly by CYP2C19. It irreversibly inhibits the P2Y12 receptor. Clinical limitations include variable response due to genetic polymorphisms (CYP2C19 loss-of-function alleles), drug interactions, and a slower onset compared with newer agents [19]. Genotype-guided strategies are commonly discussed as precision approaches.
- **Ticagrelor** is an oral cyclopentyltriazolopyrimidine that binds reversibly to P2Y12 and achieves rapid, potent inhibition without needing metabolic activation. It provides more consistent platelet inhibition than clopidogrel. Adverse drug effects include dyspnea and reversible increases in serum creatinine and uric acid [10].
- **Prasugrel** is an oral thienopyridine with more efficient and consistent bioactivation than clopidogrel. It irreversibly inhibits P2Y12. Prasugrel has greater potency and a higher bleeding risk compared with clopidogrel. It is recommended preferentially for many ACS patients undergoing PCI, unless there are contraindications, such as prior stroke/transient ischemic event [17]. A dose reduction is recommended for advanced age and low weight.
- **Cangrelor** is an intravenous, fast-acting and rapidly reversible P2Y12 inhibitor used for peri-procedural platelet inhibition when oral agents are not feasible or when rapid offset is desirable [18].
- **Selatogrel (Novel agent)** is an investigational subcutaneously injectable P2Y12 antagonist allowing rapid on-demand blockade, and early-phase data suggest utility for pre-hospital or rescue therapy, but phase III evidence is pending [21].

**Dual pathway inhibition** includes combinations of antiplatelets with low-dose anticoagulants (such as rivaroxaban 2.5 mg twice daily plus aspirin in chronic coronary disease), which have demonstrated benefit in selected high-ischemic, low-bleeding risk populations [22]. In ACS, the additive bleeding risk currently limits routine combined anticoagulant strategies outside specific indications.

## 3. Contemporary Guideline Recommendations and Risk Stratification in ACS

The 2023 ESC Guidelines for the Management of Acute Coronary Syndromes [4] provide a nuanced, risk-stratified framework for DAPT duration after ACS with PCI. A Class I recommendation (Level of Evidence A) endorses 12 months of DAPT with a potent P2Y12 inhibitor (ticagrelor or prasugrel) for most patients with ACS without prohibitive bleeding risk. However, a Class IIa recommendation supports abbreviated DAPT of 1–3 months followed by P2Y12-inhibitor monotherapy in patients at high bleeding risk (ARC-HBR criteria), supported by MASTER-DAPT and TWILIGHT data. For selected non-high-bleeding-risk patients in whom bleeding concerns are present, a Class IIb recommendation permits earlier de-escalation based on TWILIGHT and ULTIMATE-DAPT data [23,24,25]. The 2021 ACC/AHA/SCAI Coronary Revascularization Guideline similarly endorses 6–12 months DAPT for ACS patients (Class I, Level A), with de-escalation strategies in HBR patients acknowledged at Class IIb [7]. It favors ticagrelor or prasugrel plus aspirin in the absence of high bleeding risk [26,27]. For high-bleeding risk patients, short courses (3–6 months) are permitted, supported by large trials, such as STOPDAPT-2, MASTER-DAPT, and SMART-CHOICE [24,27,28]. In selected patients, guidelines endorse even earlier transition (1–3 months) to P2Y12-inhibitor monotherapy—in particular, ticagrelor—based on evidence from TWILIGHT, GLOBAL LEADERS, and STOPDAPT-2 demonstrating reduced bleeding without increased ischemic events [23,28,29]. These strategies still require careful judgment since the earliest withdrawal approaches have not been uniformly safe across all ACS presentations.

The guidelines also encourage clinicians to use structured tools to judge bleeding and ischemic risk rather than relying on instinct alone. The PRECISE-DAPT [30] and DAPT [31] scores are the most commonly used instruments. They draw on simple variables, such as age, renal function, hemoglobin, white cell count, and any prior history of bleeding, to estimate a patient’s risk of harm from longer DAPT. Their purpose is not to dictate decisions but to support a clearer, documented rationale for shortening or extending therapy. Observational studies suggest that patients with high PRECISE-DAPT scores, typically 25 or above, experience more harm than benefit from maintaining longer therapy, which strengthens the argument for earlier de-escalation in this group.

Most recently, the 2025 ACC/AHA/ACEP/NAEMSP/SCAI guideline for the management of patients with acute coronary syndromes has formally endorsed P2Y12-inhibitor monotherapy as a Class IIa recommendation following at least one month of DAPT, with aspirin discontinuation during the maintenance phase considered acceptable in appropriate patients [32]. Both ticagrelor and prasugrel retain Class I recommendations as the preferred P2Y12 inhibitors in ACS, consistent with the accumulating trial evidence reviewed herein. This guideline update represents the first major American guideline to formally sanction a shorter DAPT approach in ACS, closing the gap with the 2023 ESC guidelines.

Several emerging prediction models incorporate machine-learning approaches and biomarker integration, including troponin, D-dimer, and platelet-related lipid signatures [33,34]. These strategies may ultimately refine risk discrimination beyond conventional clinical scores. However, they remain exploratory and are not yet validated for routine decision-making in ACS.

Overall, the shift in guidelines and risk stratification supports a more flexible approach to antiplatelet therapy after ACS. The decision is less about rigid time frames and more about understanding how each patient’s bleeding and thrombotic risks evolve in the months following PCI.

## 4. Evidence for Abbreviated DAPT and De-Escalation Strategies After ACS: Randomized Controlled Trials and Meta-Analyses (2023–2025)

The recent literature on antiplatelet therapy after ACS reflects a shift away from a uniform 12-month model toward a more flexible, risk-responsive strategy. Before reviewing individual trials, an important methodological caveat must be emphasized. A substantial proportion of the key trials in this field—including T-PASS [35] (100% Korean), STOPDAPT-2 ACS [26] and STOPDAPT-3 [36] (100% Japanese), 4D-ACS [37] and SMART-CHOICE [27] (predominantly Korean), HOST-EXAM [38], and HOST-REDUCE-POLYTECH-ACS 2 [39] (100% Korean)—were conducted exclusively or predominantly in East Asian populations. East Asian individuals have higher CYP2C19 loss-of-function allele prevalence (approximately 60% in Japanese compared with approximately 30% in Europeans), lower average body weight, and distinct baseline bleeding risk profiles [40,41]. Consequently, the magnitude of bleeding reduction observed with abbreviated DAPT in these populations may not translate directly to Western or other non-East-Asian cohorts. This caveat applies particularly to trials demonstrating superiority for bleeding endpoints and should be considered throughout the evidence synthesis below.

The Academic Research Consortium has clarified that de-escalation covers three mechanistically distinct methods: stopping one antiplatelet agent, switching to a less potent P2Y12 inhibitor, and reducing the dose of an existing agent [42]. These categories behave differently because the biological hazards are not symmetrical. Thrombotic risk peaks in the early days after PCI. Bleeding risk develops more gradually and is dominated by spontaneous events after hospital discharge. This temporal imbalance helps explain why ultra-early aspirin withdrawal often performs poorly, while one-month or three-month strategies achieve a safer trade-off.

**Figure 1:**
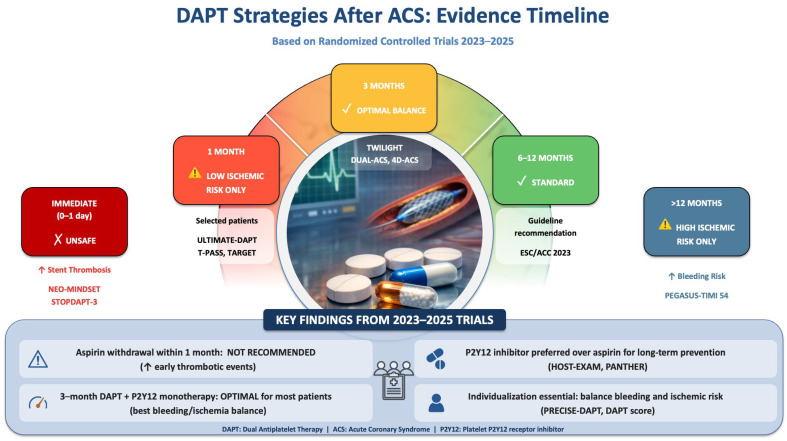
Contemporary dual antiplatelet therapy de-escalation strategies after acute coronary syndrome. Conceptual overview of dual antiplatelet therapy (DAPT) de-escalation strategies after acute coronary syndrome (ACS), illustrating the timing of aspirin withdrawal, transition to P2Y12-inhibitor monotherapy, and associated ischemic and bleeding risk trade-offs across the post-PCI period. Ultra-early aspirin withdrawal is associated with excess thrombotic risk, whereas abbreviated DAPT followed by potent P2Y12-inhibitor monotherapy after 1–3 months provides a more favorable balance in selected patient.

The trials discussed in this section are summarized in Table 1, which provides comparative data on study populations, withdrawal timing, monotherapy agents, and key bleeding and ischemic outcomes across the spectrum of abbreviated DAPT strategies. Figure 1 summarizes the timing, mechanism, and clinical trade-offs of contemporary DAPT de-escalation strategies evaluated in recent randomized trials.

### 4.1. Aspirin Withdrawal in the First Few Days After PCI

A smaller body of work has examined whether aspirin can be withdrawn within the first few days after PCI. A single-arm prasugrel monotherapy pilot study in low-risk CCS ceased aspirin at PCI and showed acceptable safety, but its findings do not extend meaningfully to ACS practice [43].

The most informative evidence now comes from randomized studies in ACS. In NEO-MINDSET, immediate monotherapy with ticagrelor or prasugrel led to fewer major or clinically relevant bleeds but more early ischemic events, including a higher number of stent thromboses. It failed to meet the prespecified non-inferiority criterion for the primary composite endpoint (all-cause death, MI, definite or probable stent thrombosis, stroke, or urgent revascularization at 30 days; HR 1.28; 95% CI 0.98–1.68). The upper confidence interval boundary exceeded 1.0, indicating a clinically meaningful trend toward harm. Critically, the failure to meet non-inferiority (a design failure indicating insufficient evidence of safety) is distinct from a statistically significant increase in events. Both conclusions suggest immediate aspirin withdrawal in ACS is not yet established as safe [44]. STOPDAPT-3 reached comparable conclusions. Prasugrel monotherapy from PCI did not reduce bleeding and produced a small but clinically meaningful excess of stent thrombosis within the first 30 days [36].

Taken together, these trials suggest that the earliest post-PCI period in ACS remains too vulnerable for routine aspirin-free strategies, even with potent P2Y12 inhibition.

The excess thrombotic events observed with immediate aspirin withdrawal raised the question of whether a brief period of dual therapy might provide adequate early protection while still permitting earlier de-escalation than conventional strategies.

### 4.2. Very Early Aspirin Withdrawal at One Month

Several trials have evaluated whether aspirin can be withdrawn after a brief one-month period of DAPT.

ULTIMATE-DAPT [25] and T-PASS [35] both showed that one-month DAPT followed by ticagrelor monotherapy reduced bleeding without a clear increase in MI, stroke or stent thrombosis. TARGET-FIRST, in low-risk MI patients with complete revascularization, produced similar findings and supported the feasibility of early transition in carefully selected groups [45].

GLOBAL LEADERS, although larger and designed around a one-month DAPT run-in before ticagrelor monotherapy, did not demonstrate superiority for their primary endpoint and showed no clear early bleeding benefit [29]. However, its one-month aspirin-withdrawal design places it in this category rather than among immediate-withdrawal studies. Its neutral findings serve mainly to emphasize the heterogeneity of one-month strategies, especially when event-free run-in and population risk differ.

STOPDAPT-3, while primarily testing immediate withdrawal, indirectly reinforces the principle that protection in the first month is critical [36]. In contrast, when one month of full DAPT is completed, as in ULTIMATE-DAPT, T-PASS and TARGET-FIRST, results are consistently more favorable. These patterns suggest that stability during the first month is a key determinant of whether early monotherapy is safe.

Complementing individual trial data, a post-hoc comparative analysis pooling individual patient data from TICO and T-PASS (*n* = 2953) further refined the optimal timing of aspirin discontinuation with ticagrelor monotherapy in ACS [46]. Patients in whom aspirin was discontinued within one month of PCI experienced significantly lower rates of BARC type 3 or 5 bleeding compared with those in whom discontinuation occurred at three months, without a significant increase in major adverse cardiovascular events. These data directly support earlier aspirin withdrawal—within one month—as the preferred timing when ticagrelor monotherapy is the intended strategy.

While one-month DAPT strategies demonstrated variable success depending on patient selection and P2Y12 inhibitor choice, three-month strategies emerged as a more consistently safe approach across broader populations.

### 4.3. Three-Month Strategies

Three-month DAPT has become one of the most convincing approaches for ACS patients who remain event-free during the early period. TWILIGHT showed that switching to ticagrelor monotherapy after three months significantly reduced BARC type 2, 3, or 5 bleeding at one year (4.0% vs. 7.1%; HR 0.56; 95% CI 0.45–0.68; *p* < 0.001)—approximately a 50% relative reduction—and did not increase MI or stroke. Benefits were reproduced across high-risk subgroups [23]. TWILIGHT enrolled a mixed ACS (~65%) and chronic coronary syndrome (~35%) population and used a three-month event-free run-in period, enriching the population for lower-risk survivors. This design limits generalizability to all-comers ACS patients. DUAL-ACS [47] extended these findings to a broader population and showed that three-month DAPT reduced bleeding without sacrificing ischemic protection.

These results support three-month DAPT as a reasonable default for many ACS patients.

The favorable outcomes observed with three-month strategies prompted further investigation into which specific P2Y12 inhibitors and patient populations were most suitable for early monotherapy.

### 4.4. Trials Defining the Limits of Early Monotherapy

STOPDAPT-2 ACS demonstrated that clopidogrel monotherapy begun at one to two months increased MI, despite lowering bleeding [26]. The variability in clopidogrel metabolism, present in up to one-third of patients, is amplified in ACS and contributes to inconsistent performance outside East Asian populations. STOPDAPT-3 similarly highlighted the hazards of reducing platelet inhibition too soon. In this trial, prasugrel 3.75 mg monotherapy initiated at the time of PCI did not reduce bleeding and was associated with an early excess of stent thrombosis [36]. It is important to recognize that the 3.75 mg prasugrel dose reflects Japanese-approved dosing, which differs from Western regimens and is used in a population with distinct pharmacogenomic and anthropometric characteristics [36]. While the biological principle remains consistent—namely that the immediate post-PCI phase in ACS requires robust dual-pathway platelet inhibition—extrapolation of these findings to non-East-Asian populations should be undertaken cautiously. Collectively, these data reinforce that both timing and effective antiplatelet potency are critical determinants of early monotherapy safety.

### 4.5. Tailored De-Escalation Approaches

Several studies have evaluated more measured strategies. The 4D-ACS trial [37] combined early aspirin withdrawal with prasugrel dose reduction and observed fewer bleeding events with preserved ischemic safety. HOST-REDUCE-POLYTECH-ACS 2 [39] demonstrated that structured prasugrel dose reduction after an initial standard phase lowered bleeding without increasing thrombotic events. OPT-BIRISK [48] tested prolonged clopidogrel monotherapy in patients with simultaneous high bleeding and ischemic risk and observed net clinical benefit, suggesting that extended monotherapy may be appropriate in specific dual-risk groups.

Beyond standard PCI populations, specific clinical scenarios, including coronary artery bypass grafting, presented distinct questions regarding optimal antiplatelet therapy duration and intensity.

To contextualize findings from individual trials with varying designs and populations, several meta-analytic efforts have synthesized the expanding abbreviated DAPT literature.

### 4.6. CABG-Specific Evidence

In the post-CABG setting, TOP-CABG found that three months of DAPT followed by aspirin monotherapy preserved graft patency and lowered bleeding risk [49]. TACSI, in contrast, showed that adding ticagrelor to aspirin increased major bleeding without clear clinical benefit in ACS patients [50]. These findings support aspirin monotherapy for most patients after CABG.

### 4.7. Meta-Analytic Evidence

Meta-analytic work consistently shows that early P2Y12 inhibitor monotherapy after a short DAPT course reduces bleeding without increasing composite ischemic risk in the average patient [51,52]. The PANTHER analysis demonstrated that P2Y12 inhibitor monotherapy lowers cardiovascular death, MI and stroke compared with aspirin monotherapy, with similar major bleeding [53]. Other pooled analyses suggest that ticagrelor-based monotherapy and prasugrel dose modulation are the most reliable strategies, while clopidogrel monotherapy remains more variable due to genetic and metabolic heterogeneity.

Taken together, the individual trial data and pooled analyses permit several overarching conclusions about the safety and efficacy of abbreviated DAPT strategies across the spectrum of ACS presentations.

### 4.8. Interpretation of the Evidence

Across the full range of ACS trials, a consistent message emerges (Table 1). The first days and weeks after PCI carry the greatest thrombotic hazard. Aspirin withdrawal during this ultra-early period has not proven safe. Once the initial month has passed without complications, early transition to monotherapy, most reliably with ticagrelor, is reasonable in many patients. Three-month strategies remain the most widely supported balance of efficacy and safety. Dose modulation and biologically guided transitions offer a promising route for personalization. The developing evidence supports a shift from rigid timelines to tailored strategies that reflect each patient’s ischemic risk, bleeding propensity and capacity to tolerate potent platelet inhibition.

### 4.9. Methodological Considerations and Evidence Hierarchy

Interpretation of abbreviated DAPT trials cannot rely solely on headline hazard ratios. Trial architecture influences interpretation, sometimes more than the point estimate itself. Non-inferiority margins, open-label design, and event-free run-in periods shape what these studies demonstrate and, just as importantly, what they cannot establish.

Several contemporary trials were constructed as non-inferiority studies, where the pre-specified margin defines how much excess ischaemic risk is considered acceptable in exchange for bleeding reduction. In NEO-MINDSET, the absolute non-inferiority margin was 1.5 percentage points, and the primary composite endpoint yielded a hazard ratio of 1.28 (95% CI 0.98–1.68), failing to meet the prespecified criterion for non-inferiority [44]. Failure to meet non-inferiority does not prove harm, yet the upper confidence boundary extended into a clinically concerning range. This observation aligns with the recognized early concentration of thrombotic risk following ACS, as reflected in contemporary guideline recommendations [4].

T-PASS adopted a wider 2.0 percentage-point non-inferiority margin [35]. While the trial met its primary endpoint, interpretation inevitably depends on whether such a margin is considered clinically acceptable in current practice. Non-inferiority thresholds embed clinical judgement. A strategy deemed acceptable under one margin may appear less reassuring under another.

Study structure further influences evidentiary strength. Several abbreviated DAPT trials, including NEO-MINDSET, STOPDAPT-3, T-PASS, DUAL-ACS, and 4D-ACS, were conducted in open-label fashion [35,36,37,44]. Although blinded endpoint adjudication mitigates detection bias, treatment awareness may influence clinician-driven outcomes, such as repeat angiography or revascularization. Bleeding endpoints incorporating BARC definitions remain standardized across trials, yet ascertainment differences cannot be entirely excluded in open-label designs.

Event-free run-in periods represent another important design feature. TWILIGHT randomized patients only after three months of uneventful DAPT [23], thereby excluding those with early complications. Similar enrichment strategies were applied in T-PASS [35]. This approach strengthens internal validity for the de-escalation phase but narrows generalisability. The highest-risk early window is, by design, filtered out before randomization.

Viewed collectively, the evidence forms a gradient rather than a binary distinction between safe and unsafe strategies. Three-month DAPT followed by ticagrelor monotherapy is supported by the most consistent and methodologically robust data. TWILIGHT enrolled 7119 high-risk PCI patients and demonstrated a significant reduction in BARC type 2, 3, or 5 bleeding at one year with ticagrelor monotherapy compared with continued DAPT (HR 0.56; 95% CI 0.45–0.68), without excess death, myocardial infarction, or stroke [23]. These findings are supported by contemporary meta-analyses of abbreviated strategies [51,52].

ULTIMATE-DAPT, which randomized 3400 patients after one month of DAPT and uniquely employed a double-blind, placebo-controlled design, provides particularly rigorous evidence supporting ticagrelor monotherapy in selected patients [25]. The blinded structure increases confidence that bleeding reduction was not materially influenced by performance bias.

In contrast, immediate aspirin withdrawal at the time of PCI remains less secure. NEO-MINDSET and STOPDAPT-3 both signal potential early vulnerability when dual-pathway inhibition is withdrawn too soon in ACS [36,44]. Smaller or regionally concentrated studies, such as 4D-ACS, offer important insight but provide more limited certainty because of sample size and population concentration [37]. The predominance of East Asian cohorts in several abbreviated strategies warrants careful extrapolation to non-East Asian populations, particularly in light of pharmacogenomic and bleeding-risk differences [41].

Abbreviated DAPT is therefore not a single entity, but a spectrum of strategies supported by varying degrees of methodological robustness and replication. Recognizing that gradient helps align therapeutic confidence with the strength of available evidence, especially during the early biologically unstable phase after ACS.

## 5. Balancing Ischemic and Bleeding Risks

Guidelines recommend weighing up the bleeding risk when determining treatment duration, and several scoring tools have been developed to help standardize the assessment of bleeding risk. The DAPT score [31] predicts the benefit of extending DAPT beyond a year, and the PRECISE-DAPT score is a five-item score used to predict bleeding on DAPT, considering age, creatinine clearance, hemoglobin, white blood cell count, and prior spontaneous bleeding [30]. Observational data demonstrated that among high-risk patients (PRECISE-DAPT score above 25), prolonged DAPT was associated with a higher bleeding burden, but no ischemic benefit. This suggests that in the clinical scenario of a high PRECISE-DAPT score, an abbreviated period of DAPT may be more appropriate, although more prospective testing of these high-risk prediction models in the setting of RCTs is required.

**Figure 2:**
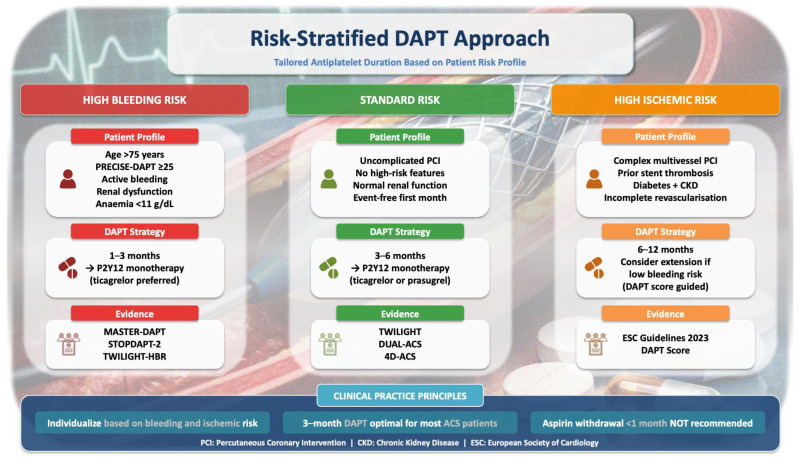
Time-dependent balance of ischemic and bleeding risk after PCI for acute coronary syndrome. Schematic representation of the dynamic relationship between ischemic and bleeding risk following percutaneous coronary intervention (PCI) for acute coronary syndrome. Ischemic risk is highest in the early post-PCI phase and declines over time, whereas bleeding risk accumulates more gradually. This temporal divergence underpins contemporary strategies favoring abbreviated DAPT followed by P2Y12-inhibitor monotherapy in appropriately selected patients.

Figure 2 illustrates a practical, time-dependent framework for integrating bleeding and ischemic risk to guide DAPT duration after ACS.

The clinical application of these risk stratification tools must be informed by trial-specific outcomes. Table 1 summarizes bleeding and ischemic endpoints across major abbreviated DAPT trials, illustrating the heterogeneity in event rates that underscores the importance of individualized risk assessment.

While traditional bleeding-risk tools, such as PRECISE-DAPT and ARC-HBR, are clinically validated, newer predictive models using machine-learning techniques have been proposed. These incorporate multidimensional variables, including biomarkers and platelet function parameters, and show promise in improving discrimination of thrombotic and bleeding risk [33,34]. However, current evidence remains observational, and prospective trials demonstrating improved clinical outcomes from algorithm-guided DAPT modification are lacking. At present, these tools should be considered investigational rather than practice-defining.

In addition, abbreviated DAPT strategies are not universally appropriate. Prolonged DAPT should be maintained in patients with low bleeding risk and particularly high ischemic burden. Clinical scenarios favoring standard or extended duration DAPT include complex left main or bifurcation disease, incomplete revascularization with high residual SYNTAX score, prior stent thrombosis despite adequate antiplatelet therapy, and extensive multivessel disease in patients with diabetes mellitus or chronic kidney disease without concurrent bleeding risk factors. In these populations, the thrombotic hazard may outweigh bleeding concerns, and individualized risk assessment remains paramount. Conversely, any intercurrent bleeding event, development of severe anemia, or acute kidney injury should prompt immediate reassessment and consideration of early DAPT discontinuation, regardless of initial risk stratification.

## 6. Special Populations

### 6.1. High Bleeding Risk (HBR) Patients

Balancing bleeding against ischemic risk when deciding on optimal DAPT duration is important, especially in certain vulnerable patient cohorts. Elderly patients over the age of 75 years old experience substantially higher rates of major bleeding after PCI [42,54]. A standardized definition of high bleeding risk (HBR) is provided by the Academic Research Consortium for High Bleeding Risk (ARC-HBR) criteria [42], which identify patients at HBR based on the presence of one major criterion (e.g., anticipated long-term oral anticoagulation, severe or end-stage CKD, hemoglobin <11 g/dL, active malignancy, prior spontaneous intracranial hemorrhage) or two minor criteria. These criteria were used as inclusion criteria for MASTER-DAPT and influenced enrolment in several other abbreviated DAPT trials. Clinicians should apply ARC-HBR criteria systematically when considering DAPT abbreviation, as they provide an objective and reproducible framework for HBR identification.

Evidence from several trials supports abbreviated DAPT in patients at high bleeding risk. The STOPDAPT-2 trial (all-comers population) demonstrated that clopidogrel monotherapy after 1–2 months of DAPT was non-inferior and superior to standard 12-month DAPT for net clinical outcomes. However, the ACS-specific sub-study, STOPDAPT-2 ACS, failed to demonstrate non-inferiority for its composite primary cardiovascular endpoint (HR 1.50; 95% CI 0.99–2.26; *p* for non-inferiority = 0.06), driven by excess MI and stent thrombosis in the clopidogrel monotherapy arm. Clinicians should therefore not extrapolate the parent STOPDAPT-2 result to ACS patients when considering clopidogrel monotherapy after abbreviated DAPT [26,28]. The TWILIGHT trial investigated three-month DAPT followed by ticagrelor monotherapy in comparison to standard DAPT therapy, focusing on high-bleeding-risk (HBR) population, including elderly individuals (mean age 65 ± 10.4 years). This found significantly reduced bleeding (HR 0.56; *p* < 0.001) without an increase in mortality or ischemic events [23]. The MASTER-DAPT trial further supported these results, where one-month DAPT duration in the HBR population achieved non-inferior results in preventing ischemic events, with a substantially lower bleeding risk (difference −2.82%; *p* < 0.001) [24]. Therefore, this suggests that in an HBR population, an abbreviated DAPT strategy of 1–3 months may be safer and effective, without compromising protection from ischemic complications.

### 6.2. Patients with High Ischemic Risk: CKD and Diabetes

Despite the data showing a lower risk of bleeding with an abbreviated DAPT strategy, caution must be applied to certain patient populations that may have a higher ischemic risk. This includes patients with chronic kidney disease (CKD) and diabetes mellitus who have inherently higher thrombotic risk and experience increased incidence of MACCE [55,56,57].

In CKD, uremic platelet dysfunction leads to a paradoxical hemostatic impairment alongside prothrombotic platelet activation, complicating the interpretation of antiplatelet efficacy. In diabetes mellitus, persistent platelet hyperreactivity, impaired CYP2C19-mediated clopidogrel activation, and high on-treatment platelet reactivity despite standard DAPT may attenuate the protective effect of antiplatelet therapy and contribute to residual ischemic risk [55,56,57]. The DAPT trial subgroup analysis in diabetic patients suggested a trend toward ischemic benefit from extended DAPT beyond 12 months (HR 0.72; 95% CI 0.54–0.97) [6]. However, this benefit was not consistently reproduced in meta-analyses of unselected populations.

Although these findings might imply a role for prolonged DAPT in these populations, multiple studies have failed to demonstrate improved outcomes with extended DAPT [57,58,59,60]. These data suggest that prolonging DAPT beyond the standard 12 months, based solely on the presence of CKD or diabetes, is not supported by the current evidence. There is also a lack of data on DAPT duration in more complex PCI procedures with higher thrombotic risk, including left main or bifurcation stenting.

### 6.3. Patients Requiring Oral Anticoagulation

In terms of patients requiring long-term oral anticoagulant therapy, such as in atrial fibrillation, combining triple therapy (aspirin, P2Y12 inhibitor and an oral anticoagulant) substantially increases bleeding risk, without a proportional reduction in thrombotic events. Direct oral anticoagulants (DOACs) are preferred over warfarin following PCI for patients with ACS and atrial fibrillation, as supported by multiple landmark RCTs, which report substantially lower rates of bleeding while maintaining comparable protection against thromboembolic events [61,62,63]. The PIONEER AF-PCI trial demonstrated that rivaroxaban-based dual therapy (rivaroxaban plus clopidogrel) significantly reduced bleeding compared with warfarin-based triple therapy (HR 0.59; 95% CI 0.47–0.76) [64]. The RE-DUAL PCI trial showed that dabigatran 110 mg or 150 mg dual therapy (with clopidogrel or ticagrelor) similarly reduced major or clinically relevant non-major bleeding vs. warfarin triple therapy (dabigatran 110 mg: HR 0.52; 95% CI 0.42–0.63; dabigatran 150 mg: HR 0.72; 95% CI 0.58–0.88), without a significant increase in thromboembolic events [62].

Both the AUGUSTUS and ENTRUST-AF PCI trials demonstrated that aspirin-free regimens, consisting of a DOAC plus a P2Y12 inhibitor, significantly reduced bleeding while preserving ischemic safety compared with traditional triple therapy [63,65]. These results underpin the current ESC and ACC/AHA guidance for early aspirin discontinuation—typically at hospital discharge or within the first week after PCI, consistent with the median discontinuation time in AUGUSTUS [4,66]. Prolonged triple therapy is now reserved only for cases with very high ischemic risk and low bleeding risk, in whom aspirin may be continued for up to one month. As potent P2Y12 inhibitors (ticagrelor and prasugrel) substantially increase bleeding when combined with oral anticoagulation, clopidogrel is the preferred agent in dual therapy regimens. Accordingly, guideline-directed therapy recommends a DOAC plus clopidogrel for up to 12 months, followed by DOAC monotherapy thereafter [4].

### 6.4. Sex-Specific Considerations

Sex-based variation in bleeding risk is increasingly recognized, though evidence remains variable. Some studies suggest women may experience higher rates of early bleeding. In the LEADERS FREE study, female patients had a greater 30-day bleeding incidence than males [67]. Similar findings were reported by Kodaira M et al. in a sex-specific analysis [40]. Despite these observations, female sex is not included in major bleeding-risk models, such as PRECISE-DAPT or the PARIS registry, as it has not consistently emerged as an independent predictor.

However, several indirect factors, such as lower body mass index, reduced creatinine clearance, heightened platelet reactivity, and smaller vascular caliber, may contribute to differential bleeding risk in women [40,68,69,70]. Sex-specific considerations may therefore be relevant when individualizing DAPT duration, although dedicated prospective trials are still lacking. Sex-stratified subgroup analysis from TWILIGHT demonstrated consistent bleeding reduction with ticagrelor monotherapy in women (HR 0.60; 95% CI 0.40–0.89) without excess ischemic events, supporting the generalizability of this strategy to female patients [23]. Similarly, the MASTER-DAPT trial showed consistent non-inferiority of abbreviated DAPT in women at high bleeding risk [24]. A practical clinical approach should therefore incorporate sex as a modifying factor within a broader individualized risk assessment framework.

## 7. Long-Term Secondary Prevention Beyond One Year

Emerging evidence increasingly supports P2Y12-inhibitor monotherapy as an effective alternative to lifelong aspirin for long-term secondary prevention. The PANTHER meta-analysis demonstrated a significant reduction in composite cardiovascular events over two years, driven largely by fewer MIs, without any significant increase in major bleeding in the P2Y12 inhibitor group (clopidogrel or ticagrelor) [53]. The HOST-EXAM extended trial showed that clopidogrel monotherapy beyond five years yielded superior net clinical outcomes compared with aspirin alone (HR 0.66; 95% CI 0.53–0.82; *p* < 0.001), while maintaining similar bleeding rates [38]. In contrast, in the PEGASUS-TIMI 54 trial, extending DAPT to a mean duration of 33 months reduced ischaemic events but at the cost of substantially higher major bleeding [71].

When considering long-term aspirin vs. P2Y12, it is important to note that CYP2C19 loss-of-function alleles are present in roughly 30% of individuals of European ancestry. These polymorphisms can lead to suboptimal platelet inhibition and higher thrombotic risk [20]. The POPular Genetics trial (*n* = 2488 patients undergoing primary PCI) offered a potential solution by randomizing patients to CYP2C19 genotype-guided P2Y12 inhibitor selection (clopidogrel for extensive or intermediate metabolizers; ticagrelor or prasugrel for poor metabolizers) vs. standard potent P2Y12 inhibitor therapy. Genotype-guided de-escalation was non-inferior for net adverse clinical events at 12 months (HR 0.78; 95% CI 0.61–1.00) and significantly reduced bleeding (HR 0.78; 95% CI 0.61–0.98), while proving cost-effective in subsequent economic analyses [20,72]. Given that approximately 30% of individuals of European ancestry and up to 60% of East Asians carry CYP2C19 loss-of-function alleles, genotype-guided de-escalation has particular relevance for longer-term monotherapy decisions and for patients with high bleeding risk in whom clopidogrel is planned as the long-term agent.

Therefore, P2Y12-inhibitor monotherapy, particularly clopidogrel, can be considered an option for long-term secondary prevention beyond one year following ACS or PCI. Genotype-guided selection may need to be considered to identify clopidogrel hypo-responsiveness in certain populations. Prolonged DAPT can reduce ischaemic risk but is generally limited by excess bleeding and should be reserved for carefully selected, high thrombotic risk cases.

## 8. DAPT After ACS in Patients Undergoing CABG

The current ACC/AHA guidelines endorse lifelong aspirin following coronary artery bypass grafting (CABG) as a Class I recommendation [7,73]. DAPT is only advised in patients with acute unstable angina undergoing urgent surgical revascularization [73]. Evidence specifically addressing optimal DAPT duration after CABG remains limited. Mechanistic data suggest an elevated risk of early saphenous vein graft (SVG) stenosis within the first three months after surgery due to activated platelet function, and that DAPT during this early period may help maintain SVG patency [74,75]. However, systematic reviews consistently show that DAPT also confers a significantly higher bleeding risk compared to aspirin monotherapy, leading to increased mortality and reoperation rates [75,76,77]. The TACSI study found that DAPT doubled major bleeding risk without providing a significant improvement in clinical outcomes (HR 1.06; 95% CI 0.84–1.34; *p* = 0.63) [50]. The TOP-CABG trial evaluated a de-escalation strategy in which patients received three months of DAPT followed by aspirin alone, with a primary efficacy endpoint of graft occlusion by CT angiography at three months. Clinically relevant bleeding was significantly lower in the de-escalation group (HR 0.62; 95% CI 0.48–0.81; *p* < 0.001), while graft patency was preserved, with an absolute efficacy difference of –0.31% (p_non-inferiority_ = 0.008) [49].

Therefore, aspirin monotherapy remains adequate for most patients following CABG. A short course of DAPT (approximately three months) may be beneficial in selected high-risk individuals, such as those with multiple vein grafts, diffuse graft disease, or those requiring urgent surgical revascularization. Routine prolonged DAPT after CABG is not supported by current evidence due to increased bleeding and lack of outcome benefit.

## 9. Emerging Evidence and Future Directions

Recent evidence has begun exploring whether ultra-short DAPT (≤1 month) can balance ischemic and bleeding risk in selected patients. Early aspirin withdrawal in low-risk patients revascularized for acute MI, who completed an uncomplicated 1-month course of DAPT, reduces bleeding risk without compromising ischemic protection [45]. A 2025 meta-analysis of RCTs that compared ultra-short-term DAPT (≤1 month) compared to standard therapy (≥6 months) after DES implantation observed a 20% reduction in net adverse clinical events (NACE) and nearly 50% reduction in clinically relevant bleeding vs. standard-duration DAPT, while ischemic events remained similar. This occurred irrespective of the P2Y12 receptor antagonist choice (clopidogrel or ticagrelor) [78].

However, the generalizability of these promising findings requires careful scrutiny. Most trials supporting ultra-short DAPT were conducted in predominantly East-Asian, largely male populations. Ethnicity-subgroup data suggest that patients of East Asian ancestry, who have been historically at higher bleeding risk and lower ischemic risk, derive disproportionately greater benefit from shorter DAPT durations. A 2025 meta-analysis found that shorter DAPT significantly reduced bleeding in East-Asian cohorts (RR 0.45; 95% CI 0.34–0.59) [41,79], whereas the same bleeding reduction was not significant in non-East-Asian cohorts. Notably, there was no statistically significant difference in MI, stent thrombosis, or mortality between short and standard DAPT regardless of ethnicity, findings that were supported by another meta-analysis [78]. Therefore, routine application of ultra-short DAPT is still premature and requires more representative data.

Several other investigational approaches may also challenge the landscape of antithrombotic therapy. The novel P2Y12 antagonist, selatogrel, is now in phase three evaluation. It offers rapid onset and favorable safety with potential for pre-hospital or self-administered ACS treatment [80]. Other emerging agents, such as BMS-986141 (a PAR-4 inhibitor), are in phase 1 and 2 trials in patients with stable coronary artery disease. BMS-986141 provided significant, dose-dependent inhibition of PAR-4–triggered platelet aggregation when added to background antiplatelet therapy [81]. Preclinical and translational studies also show that combining PAR-4 antagonism with very-low-dose factor Xa inhibition produced additive antithrombotic effects without disproportionate bleeding, supporting the possibility of dual-pathway inhibition [82].

Finally, precision-medicine tools may help personalize therapy in the future. Pharmacogenomic profiling may become more relevant as P2Y12-monotherapy strategies expand. The use of platelet function testing to guide predicted thrombotic risk and monitor individual response to antiplatelet therapy has been preliminarily investigated. However, current studies have not shown that treatment modification, based on platelet function test results, has improved the outcomes [83]. Further prospective investigation is required to determine whether these approaches can be integrated into routine clinical practice.

Regarding platelet function testing, its clinical utility is further limited by timing dependency (results vary significantly based on when testing is performed relative to drug administration and clinical state) [84], analytical variability across platforms (VerifyNow, Multiplate, PFA-100) [85,86], and biological confounders, including anemia, renal impairment, and inflammatory state [87,88,89]. Until prospective outcome trials validate a specific testing-guided algorithm, platelet function testing should not be used to routinely guide DAPT duration decisions.

Optimal PCI techniques, including adequate lesion preparation, appropriate stent sizing, complete expansion confirmed by intracoronary imaging, and avoidance of stent mal-apposition, are critical in determining late outcomes after PCI. This should be considered alongside DAPT duration decisions. Newer drug-eluting stent platforms with ultrathin struts and bioresorbable polymers (e.g., BIOFREEDOM, Synergy, and ONYX ONE) have lower thrombogenicity profiles than earlier-generation stents and may independently support earlier DAPT abbreviation. This is evidenced by the LEADERS FREE [90] and ONYX ONE [91] trials in high-bleeding-risk populations. These platform advances complement but do not replace clinical risk stratification when determining DAPT duration.

An emerging area of importance is the role of intravascular imaging in optimization PCI technique. Imaging-guided PCI—using optical coherence tomography (OCT) or intravascular ultrasound (IVUS)—optimizes stent expansion, minimizes mal-apposition, and reduces edge dissection, thereby lowering the procedural substrate for stent thrombosis. The OCTOBER trial demonstrated that OCT-guided PCI significantly reduced major adverse cardiovascular events at two years compared with angiography-guided PCI in patients with complex bifurcation lesions [92]. ILUMIEN IV showed that OCT guidance improved the minimum stent area and reduced target lesion failure [93]. These data suggest that high-quality, imaging-guided PCI may independently support safer early DAPT by reducing procedure-related thrombotic risk.

The cost-effectiveness of abbreviated DAPT strategies deserves acknowledgement. Single antiplatelet therapy after abbreviated DAPT would ultimately reduce total treatment costs by lowering hospitalization for bleeding, without increasing expenditure on ischaemic event management. The POPular Genetics trial also demonstrated the cost-effectiveness of genotype-guided de-escalation compared with universal potent P2Y12 inhibition [72].

Real-world adherence to 12-month DAPT is historically suboptimal, with premature discontinuation rates ranging from 10% to 25% [94,95,96], depending on population and healthcare context. Abbreviated DAPT strategies align more closely with observed real-world adherence patterns, potentially reducing the gap between guideline-recommended and actual therapy duration, and minimizing the clinical harm associated with unplanned therapy interruption during the highest-risk early post-PCI period.

## 10. Conclusions: Applying New Evidence in Current Clinical Practice

It is important to emphasize that antiplatelet therapy decisions occur within a broader framework of comprehensive secondary prevention after ACS. DAPT duration is only one component of a broader strategy that includes high-intensity statin therapy, renin–angiotensin system inhibition, beta-blockade where indicated, structured cardiac rehabilitation, lifestyle modification, and rigorous control of cardiovascular risk factors. Antiplatelet therapy should be reassessed at each follow-up visit in the context of the patient’s evolving risk profile. DAPT (aspirin plus a potent P2Y12 inhibitor) remains the cornerstone for antithrombotic therapy after PCI for ACS. Current international guidelines recommend 6–12 months of DAPT, with the default duration being 12 months for most patients [4]. This reflects consistent evidence demonstrating that DAPT reduces early stent thrombosis, recurrent myocardial infarction, and target-vessel revascularization, particularly during the high-risk immediate post-PCI phase.

In patients undergoing CABG, aspirin monotherapy remains the standard approach, with short-duration DAPT reserved for carefully selected high graft-related risk profiles.

Beyond one-year, prolonged DAPT reduces ischemic events but consistently increases major bleeding. In stable patients who complete 12 months uneventfully, P2Y12-inhibitor monotherapy—particularly clopidogrel, where appropriate—represents a reasonable alternative to lifelong aspirin, with pharmacogenomic considerations relevant in selected populations.

Antiplatelet duration after ACS should be individualized, integrating validated bleeding-risk frameworks, procedural complexity, and evolving patient-specific factors. While pharmacogenomic profiling and advanced risk modeling may enhance future precision, current decision-making must rely on structured clinical assessment and careful longitudinal reassessment.

Finally, abbreviated DAPT is no longer a niche strategy but a legitimate, evidence-supported option in appropriately selected patients—provided that the early post-PCI thrombotic phase is respected, and residual ischemic risk remains adequately protected.

## Figures and Tables

**Table 1:** Summary of Recent Randomized Controlled Trials (and PANTHER Meta-Analysis) of Abbreviated DAPT Strategies After Acute Coronary Syndrome. Trials are organized by the timing of aspirin withdrawal or the DAPT abbreviation strategy.

Trial	*n*	Population	Population/Geography	DAPT Duration	Monotherapy Agent	Bleeding Definition	Bleeding Outcome	Ischemic Outcome	Key Finding
NEO-MINDSET	3410	ACS (STEMI 62%)	European multicenter	Immediate	Prasugrel/ticagrelor	BARC 2–5	BARC 2–5: 2.0% vs. 4.9%, HR 0.40 (0.26–0.59)	Primary endpoint: 7.0% vs. 5.5%, HR 1.28 (0.98–1.68), NI not met	Increased early ischemic events with immediate withdrawal
STOPDAPT-3	5966	ACS or HBR	Japanese (100%)	Immediate	Prasugrel	BARC 3 or 5	BARC 3/5: 4.47% vs. 4.71%, HR 0.95 (0.75–1.20), NS	CV events: 4.12% vs. 3.69%, HR 1.12 (0.87–1.45), NI met	No superiority for bleeding; excess stent thrombosis
STOPDAPT 2 ACS	4136	ACS (STEMI 56%)	Japanese (100%)	1–2 months	Clopidogrel	TIMI major or minor	Major: 1.1% vs. 1.2%, HR 0.46 (0.23–0.94)	CV events: 2.8% vs. 1.9%, HR 1.50 (0.99–2.26), NI not met	Failed to show NI for net clinical benefit
ULTIMATE DAPT	3400	ACS	Chinese (100%)	1 month	Ticagrelor	BARC 2–5	Clinically relevant: 2.1% vs. 4.6%, HR 0.45 (0.30–0.66)	MACCE: 3.6% vs. 3.7%, HR 0.98 (0.69–1.39), NI met	Reduced bleeding without increased ischemic events
T-PASS	2850	ACS (STEMI 40%)	Korean (100%)	<1 month (mean 16 days)	Ticagrelor	BARC 3 or 5	BARC 3/5: 1.2% vs. 3.4%, HR 0.35 (0.20–0.61)	Net events: 2.8% vs. 5.2%, HR 0.54 (0.37–0.80)	Superior to standard 12-month DAPT
TARGET-FIRST	1942	Low-risk acute MI	European multicenter	1 month	P2Y12 inhibitor	BARC 2, 3, or 5	BARC 2/3/5: 2.6% vs. 5.6%, HR 0.46 (0.29–0.75)	Primary composite: 2.1% vs. 2.2%, NI met	NI for ischemic events with reduced bleeding
DUAL-ACS	5052	Type 1 MI	European multicenter	3 months	Clopidogrel/prasugrel/ticagrelor	TIMI major	Major: 3.2% vs. 4.0%, HR 0.78 (0.58–1.06)	Mortality: 2.7% vs. 3.4%, HR 0.78 (0.57–1.07)	Trend toward lower mortality and bleeding
4D-ACS	1370	ACS	Korean	1 month	Prasugrel 5 mg	BARC 2–5	BARC 2–5: 0.6% vs. 4.6%, HR 0.13 (0.03–0.58)	Composite: 4.9% vs. 8.8%, NI met	77% reduction in bleeding with dose de-escalation
OPT-BIRISK	7758	High ischemic and bleeding risk	Chinese (100%)	Extended (after 12 months)	Clopidogrel	BARC 2, 3, or 5	BARC 2/3/5: 2.5% vs. 3.3%, HR 0.75 (0.57–0.97)	MACCE: 2.6% vs. 3.5%, HR 0.74 (0.57–0.96)	Benefit in dual high-risk population
TOP-CABG	2290	CABG	Chinese (100%)	3 months	Aspirin	BARC ≥ 2	BARC ≥ 2: 8.26% vs. 13.19%, HR 0.62 (0.48–0.81)	Graft occlusion: 10.79% vs. 11.19%, NI met	De-escalation preserved graft patency
TACSI	2201	ACS requiring CABG	International	Immediate post-CABG	Aspirin	Major bleeding	Major: 2.0% vs. 4.9%, HR 2.5 (1.52–4.11)	MACE: 4.6% vs. 4.8%, HR 1.09 (0.74–1.6)	DAPT increased bleeding without ischemic benefit
Meta-analyses									
PANTHER (meta-analysis)	24,325	CAD (60.6% ACS)	International (meta-analysis)	Variable	P2Y12 inhibitor	Variable	Major: HR 0.87 (0.7–1.09), NS	CV death/MI/stroke: HR 0.88 (0.79–0.97)	P2Y12 monotherapy superior to aspirin for secondary prevention

ACS, acute coronary syndrome; BARC, Bleeding Academic Research Consortium; CABG, coronary artery bypass grafting; CAD, coronary artery disease; CV, cardiovascular; DAPT, dual antiplatelet therapy; HBR, high bleeding risk; HR, hazard ratio; MACE, major adverse cardiovascular events; MACCE, major adverse cardiovascular and cerebrovascular events; MI, myocardial infarction; NI, non-inferiority; NS, not significant; and STEMI, ST-segment elevation myocardial infarction.

## Data Availability

No new data were created or analyzed in this study. Data sharing does not apply to this article.
